# Identification of a Novel VLDLR Variant in the First Report of CAMRQ1 From Africa: Expanding the Spectrum of Cerebellar Ataxia Syndromes

**DOI:** 10.1155/humu/4661238

**Published:** 2026-04-27

**Authors:** Aseel A. Jawabri, Ainara Salazar-Villacorta, Henriette Senghor, Rokhaya Ndiaye, Alia Al-Mehrzi, Amadou Gallo Diop, Moustapha Ndiaye, Bassam R. Ali, Pedro M. Rodriguez Cruz

**Affiliations:** ^1^ Department of Genetics and Genomics, College of Medicine and Health Sciences, United Arab Emirates University, Al Ain, Abu Dhabi, UAE, uaeu.ac.ae; ^2^ Department of Neuromuscular Diseases, Institute of Neurology, University College London, London, UK, ucl.ac.uk; ^3^ Department of Neurology, Great Ormond Street Hospital and Department of Developmental Neurosciences, University College London (UCL), London, UK, ucl.ac.uk; ^4^ Neurology Department, Centre Hospitalier National Universitaire de Fann, Dakar, Senegal, chnu-fann.com; ^5^ Division of Human Genetics, Faculty of Medicine, Pharmacy and Odonto-Stomatology, University Cheikh Anta Diop, Dakar, Senegal; ^6^ ASPIRE Precision Medicine Research Institute, United Arab Emirates University, Al Ain, Abu Dhabi, UAE, uaeu.ac.ae

**Keywords:** CAMRQ1, disequilibrium syndrome (DES), endoplasmic reticulum associated degradation (ERAD), reelin signaling, very low-density lipoprotein receptor (VLDLR)

## Abstract

Cerebellar ataxia, mental retardation, and disequilibrium syndrome (CAMRQ)‐related disorders are rare, nonprogressive, autosomal recessive conditions primarily characterized by cerebellar ataxia, hypotonia, intellectual disability, delayed ambulation, and, in some cases, quadrupedal locomotion. Pathogenic variants in four disease genes, *VLDLR*, *CA8*, *WRD81*, and *ATP8A2,* have been linked to these disorders, with cases reported across various ethnic groups and geographic regions. However, no reports of CAMRQ1 (OMIM #224050) have been previously made from Africa. In this study, we report the first African family with four affected siblings exhibiting typical CAMRQ1 clinical features with varying levels of phenotypic severity. Genetic analysis revealed a novel missense homozygous variant (c.1694C > A; p.P565Q) in the *VLDLR* gene in all the affected individuals, with the parents being heterozygous. Biochemical analysis, including immunofluorescence and confocal laser microscopy, western blot, and endoglycosidase H sensitivity and resistance assay, demonstrated the retention of the p.(P565Q) VLDLR protein in the endoplasmic reticulum (ER), impairing its trafficking to the plasma membrane and thus confirming its pathogenic impact. This ER retention is expected to disrupt VLDLR‐mediated signaling pathways, including reelin signaling, thereby affecting neuronal migration. Furthermore, due to its ER retention, the p.(P565Q) is expected to induce ER stress and activate the endoplasmic reticulum‐associated degradation (ERAD) pathway. Our findings expand the genetic and geographical spectrum of CAMRQ1 and provide further functional insights into its underlying pathogenesis.

## 1. Introduction

The Cerebellar ataxia, mental retardation, and disequilibrium syndrome (CAMRQ) encompasses a group of very rare nonprogressive cerebellar disorders characterized by ataxia associated with delayed ambulation, intellectual disability, speech delay, and cerebellar hypoplasia [1]. Quadrupedal gait, although not universal, is highly characteristic of the condition [2]. Additional features, including hypotonia, lack of coordination, seizures, dysarthria, strabismus, short stature, and pes planus [3, 4], overlap with many other neurodevelopmental disorders, creating diagnostic challenges in the absence of molecular testing. These conditions were also collectively referred to as disequilibrium syndrome (DES).

CAMRQ‐related disorders are categorized into four main subtypes (CAMRQ1‐4), depending on the mutated gene: *VLDLR*, *WDR81*, *CA8*, and *ATP8A2*, respectively (Figure 1a; Tables S1 and S2) [3, 5–7]. Anecdotal associations of quadrupedal gait with pathogenic variants in *TUBB2B* and *GRID2* have been reported [8, 9] but remain unconfirmed. Despite recognizable neuroimaging features, genotype–phenotype correlation in CAMRQ is limited, and clinical features rarely allow confident attribution to a specific subtype. As a result, molecular diagnosis is essential for accurate diagnosis and for distinguishing CAMRQ from phenotypically overlapping conditions.

Figure 1(a) Relative proportion of CAMRQ‐related disorders reported to date grouped by disease‐causing gene. Data to generate Figure 1a are presented in Table S2. (b) Geographical distribution of CAMRQ‐related disorders reported to date, including the pedigree here described. (c) Clinical photographs of affected individuals from the present family demonstrating quadrupedal gait: Individual III:6 shown in the left panel, Individual III:1 in the central panels, and Individual III:4 in the right panel.

**Figure:**
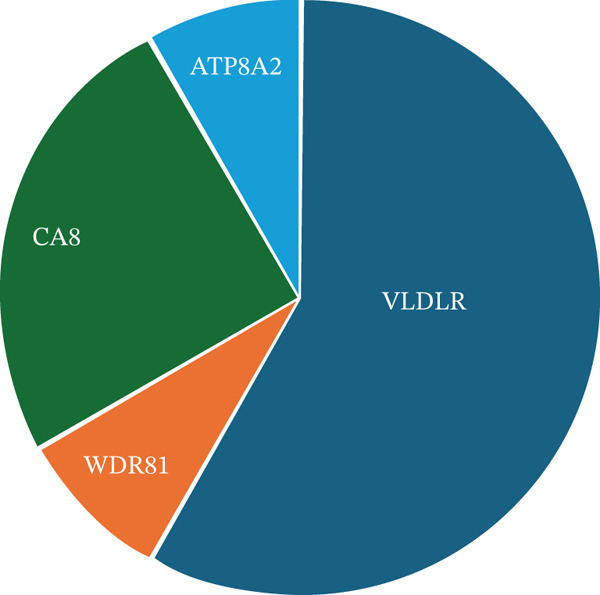
(a)

**Figure:**
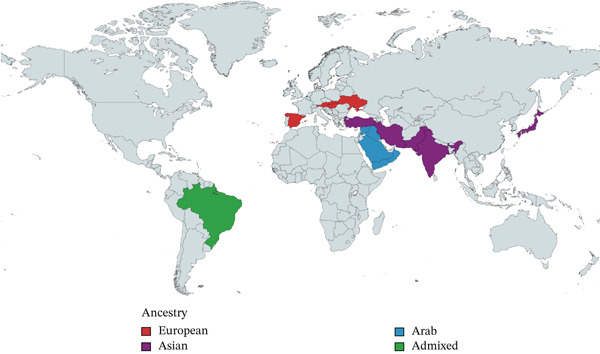
(b)

**Figure:**
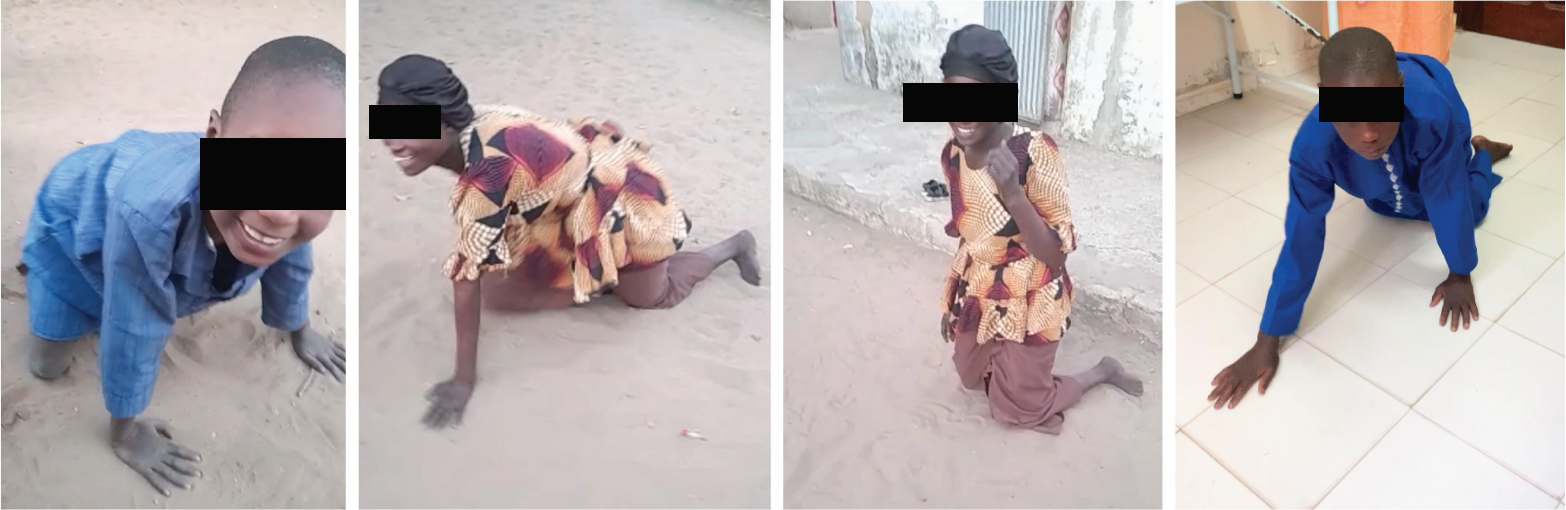
(c)

CAMRQ‐related disorders are generally very rare, with cases described mainly in the Middle East and, to a lesser extent, in Europe and East Asia (Figure 1b) [2, 10–13]. However, no genetically confirmed cases have been reported from the African continent. The absence of documented cases limits clinical suspicion; in addition, the scarcity of population‐specific reference databases makes variant interpretation particularly difficult, making functional validation crucial to reach a definitive diagnosis.

*VLDLR* encodes the very low‐density‐lipoprotein receptor, a member of the low‐density lipoprotein receptor family (Figure 2a) [15]. VLDLR is expressed in the brain, adipose tissue, skeletal muscles, heart, and endothelial cells in arterioles and capillaries, but not in the intestine or liver [16–20]. VLDLR and ApoER2/LRP8 are crucial for neuronal migration, brain layering, and synaptic plasticity [21] by binding to reelin, a large extracellular protein (> 400 kDa) involved in neurogenesis [22–24]. During embryonic development, reelin is highly expressed in Cajal‐Retzius and Purkinje cells, initiating a signaling cascade crucial for neuronal migration [18] and the “inside out” organization of the neocortex and cerebellum [3, 25, 26]. Additionally, VLDLR plays a major role in delivering triglycerides from the liver to extrahepatic tissues for hydrolysis into free fatty acids by lipoprotein lipase [15, 27]. Loss‐of‐function variants in *VLDLR* that disrupt reelin signaling have been associated with CAMRQ1, lissencephaly, epilepsy, autism spectrum disorder, and neurodegenerative disorders [26, 28, 29].

Figure 2(a) A schematic diagram of VLDLR′s structural domains, each represented in a different color and labeled with some VLDLR variants associated with CAMRQ1, including novel missense variant p.(P565Q) labeled in red and other reported VLDLR variants labeled in black [14]. Starting from the N‐terminus, the first domain is the ligand binding domain (LBD) which consists of cysteine‐rich ligand binding repeats that bind to extracellular ligands, followed by EGF‐homology domain containing the *β* propeller region, an O‐linked sugars domain for posttranslational modifications, a transmembrane domain anchoring the receptor to the cell membrane, and a cytoplasmic tail at the C‐terminus to release bound extracellular ligands in the cytoplasm. VLDLR structure was created on http://Biorender.com. (b) Family pedigree illustrating the pattern of inheritance of DES in a first‐degree consanguineous family. Circles represent females, squares represent males, and highlighted circles and squares represent an affected family member. (c) Sanger sequencing chromatograms showing the segregation of the *VLDLR* variant NM_003383.5 : c.1694C > A (p.P565G) within the family. Affected individuals are homozygous for the variant, whereas unaffected family members are heterozygous carriers. (d) Predicted three‐dimensional (3D) molecular model of VLDLR protein generated using Swiss‐Model (https://swissmodel.expasy.org/). The N‐terminus (starting amino acid, Met1) and C‐terminus (ending amino acid, Ala873) are labeled. The missense variant p.(P565Q) associated with CAMRQ1 is labeled with a green sphere.

**Figure:**
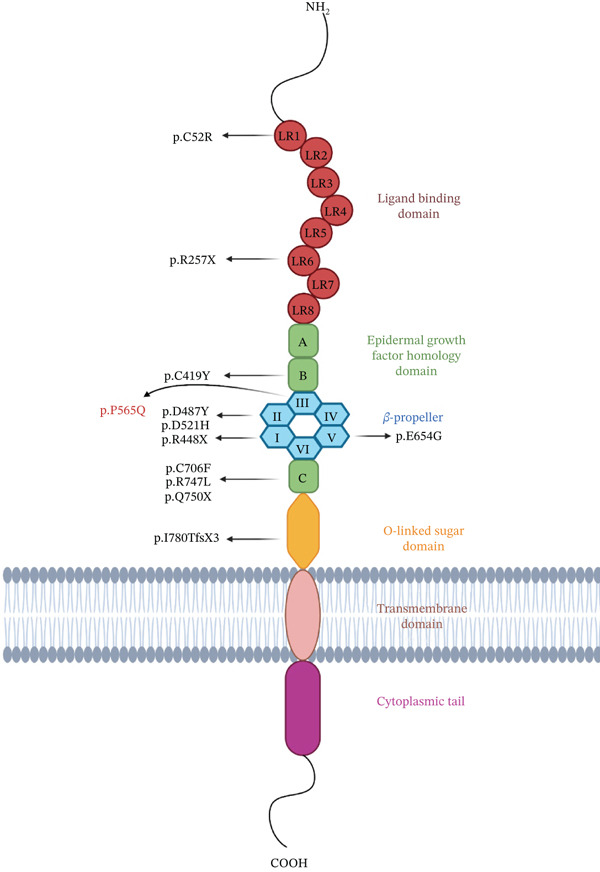
(a)

**Figure:**
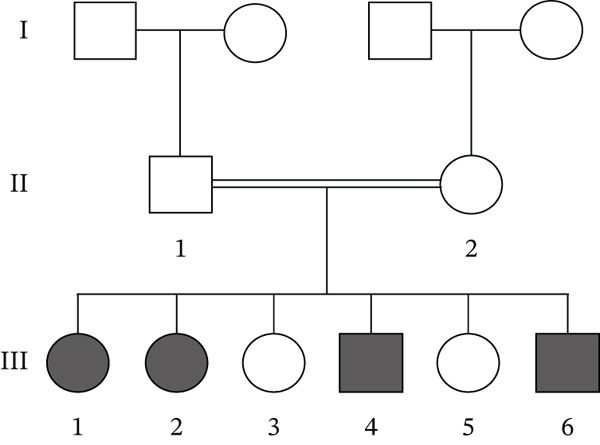
(b)

**Figure:**
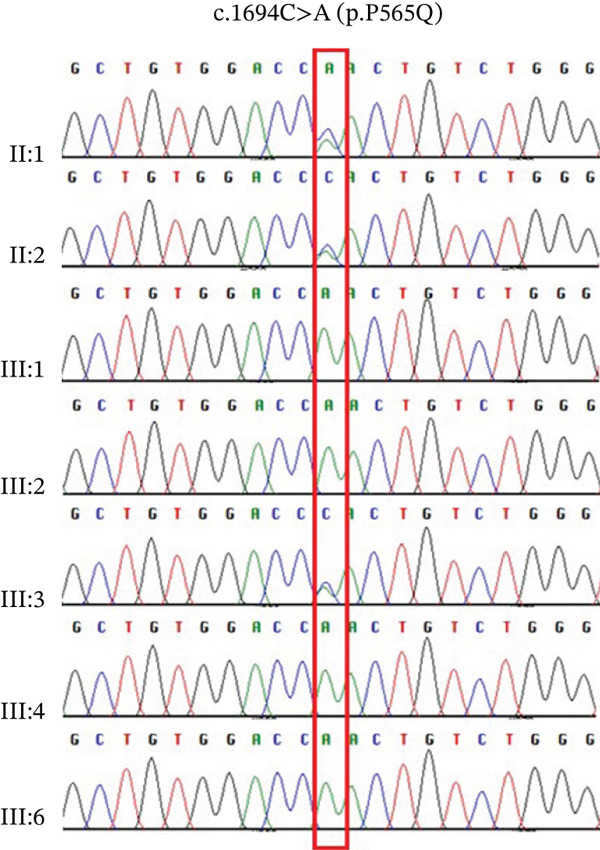
(c)

**Figure:**
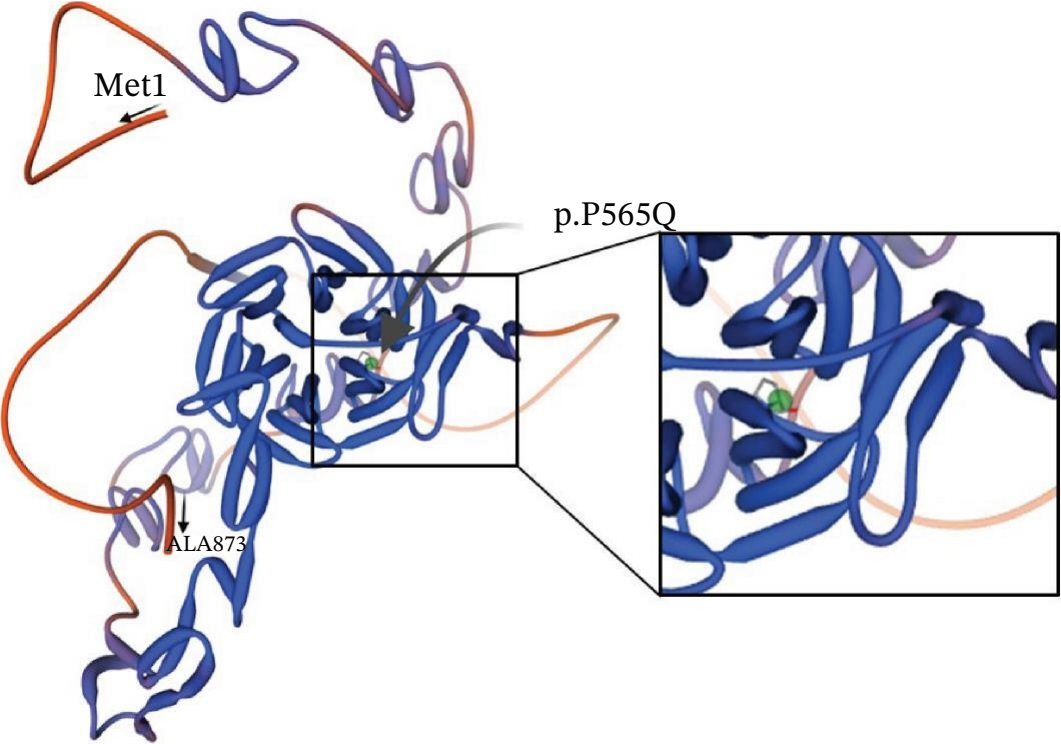
(d)

Here, we report the first description of a CAMRQ‐related disorder in the African continent and conduct functional subcellular studies to provide evidence on the pathogenicity of a novel missense variant (NM_003383.5: c.1694C > A; p.P565Q), previously classified as a variant of uncertain significance (VUS) using ACMG guidelines. Our findings provide new insight into CAMRQ1 molecular mechanisms and highlight the diagnostic value of targeted functional assays for rare neurogenetic disorders in underrepresented populations.

## 2. Materials and Methods

### 2.1. Participants

An extended consanguineous family of Wolof ethnicity with four affected siblings presenting with symptoms suggestive of disequilibrium syndrome underwent clinical and genetic studies (Figure 2b, 2c; Table 1).

**Table 1:** Clinical manifestations of CAMRQ1‐affected individuals.

General information				
Name	III:1	III:2	III:4	III:6
Age	21	19	11	9
Gender	Female	Female	Male	Male
**Pyramidal signs**				
Hyperreflexia	Yes (LLLL > UULL)	No	No	No
Spasticity	Yes (LLLL > UULL)	Yes	No	No
Babinski sign	Yes	Yes	Yes	Yes
Ankle clonus	Yes	Yes (nonsustained)	ND	ND
**Cerebellar signs**				
Ataxia	Yes	Yes	Yes	Yes
Ambulation/gait	Quadrupedal gait. Wide‐based gait with bilateral support.	Quadrupedal gait Wide‐based gait with bilateral support.	Quadrupedal gait. Wide‐based gait with bilateral support.	Quadrupedal gait. Wide‐based gait with bilateral support.
Distal hypotonia	Yes	Yes	Yes	Yes
Nystagmus	Yes	Yes	No	No
Dysdiadochokinesis	ND	ND	Yes	ND
**Ocular examination**				
Strabismus	No	Yes	Yes	Yes
**Other manifestations**				
Intellectual disability	Yes	Yes	Yes	Yes
Dysmorphism	No	Yes (mild)	No	No
Epilepsy	No	No	No	No
Short stature	No	No	No	No

Abbreviations: LLLL, lower limbs; ND, not done; UULL, upper limbs.

### 2.2. Ethics Statement

Consent for publication was obtained from all patients. Ethics approval was granted in Senegal from Comité National d′Éthique pour la Recherche en Santé (CNERS; SEN2021/26).

### 2.3. Genetic Studies

Genomic DNA was extracted from peripheral blood leukocytes. Whole exome sequencing (WES) > 90X was performed on the proband (III:1) using the KAPA HyperExome capture kit (Roche, Basels) and sequenced on Illumina NovaSeq6000 (Illumina Inc., San Diego, California) at CNAG (Barcelona, Spain). Genomic analysis was performed using the RD‐Connect Genome‐Phenome Analysis Platform [30]. Sanger sequencing was used for confirmation and segregation studies in other family members. In silico prediction tools included SIFT, PolyPhen, FATHMM, MetaLR, MetaSVM, mutation assessor, MutationTaster, and PROVEAN. Furthermore, we used DynaMut 2 [31] to investigate the effect of p.(P565Q) on VLDLR protein stability and flexibility.

### 2.4. Generation of the p.P565Q VLDLR Expression Construct

The VLDLR variant (c.1694C > A; p.(P565Q)) was generated by site‐directed mutagenesis using *PfuUltra* enzyme (Agilent, Santa Clara, United States) and mutagenic primers listed in (Table S3) designed on “PrimerX” (http://bioinformatics.org/primerx). The missense variant was introduced into a C‐terminally HA‐tagged VLDLR expression vector [32–34] and then sequenced using Applied Biosystems SeqStudio Genetic Analyzer VLDLR wild‐type (WT) transcript (NM_003383.5) was aligned with the sequence of the generated variant on Clustal Omega (https://www.ebi.ac.uk/jdispatcher/msa/clustalo) to confirm the introduction of the intended amino acid substitution within VLDLR′s sequence (Figure S1).

### 2.5. Cell Culture and Transfection

For immunofluorescence and confocal laser microscopy, HeLa cells were cultured in Dulbecco′s Modified Eagle’s Medium (DMEM; Invitrogen, Carlsbad, California, United States), supplemented with 10% fetal bovine serum (FBS; Invitrogen) and 100 U/mL penicillin/streptomycin and maintained at 37°C in 5% CO_2_ incubators. HeLa cells were seeded onto sterile coverslips in a 24‐well plate at 40%–50% confluence and were later transiently transfected with 1 *μ*g of either HA‐tagged VLDLR WT or p.(P565Q) mutant expression construct and cotransfected with 0.5 *μ*g of the plasma membrane marker GFP‐HRas plasmid using FuGENE HD (Promega, Madison, Wisconsin, United State) diluted in 25 *μ*l of Opti‐MEM Reduced Serum Medium (Gibco; Grand Island, New York, United States) and added to the cells for 24 h.

For endoglycosidase H (Endo H) sensitivity and resistance assay and western blot analysis, HEK293T cells were cultured in DMEM (Invitrogen, Carlsbad, California, United States), supplemented with 10% FBS (Invitrogen) and 100 U/mL penicillin/streptomycin, and maintained at 37°C in 5% CO2 incubators. The cells were seeded in a 6‐well plate at 40%–50% confluence and were later transiently transfected with 1 *μ*g of either HA‐tagged VLDLR WT or the VLDLR p.(P565Q) mutant expression construct diluted in 45 *μ*L of 1X Opti‐MEM Reduced Serum Medium and transiently transfected into the cells using FuGENE HD for 48 h.

To assess the degradation route of VLDLR WT and VLDLR missense variant p.(P565Q), we transiently transfected CRISPR‐Cas9‐generated HRD1 E3 ubiquitin ligase knock out (KO) cells and HEK293 (parental cell line) cultured in DMEM (Invitrogen, Carlsbad, California, United States), supplemented with 10% FBS (Invitrogen) and 100 U/mL penicillin/streptomycin with 1 *μ*g of VLDLR WT or VLDLR p.(P565Q) and incubated the cells for 48 h at 37°C in 5% CO_2_ incubators. Posttransfection, cells were harvested, and protein was extracted using the same protocol for western blot analysis.

### 2.6. Immunofluorescence and Confocal Laser Microscopy

Twenty‐four hours posttransfection, HeLa cells overexpressing VLDLR WT or VLDLR p.(P565Q) were fixed with methanol for 5 min at 4°C and subsequently blocked with 1% Bovine Serum Albumin (BSA) at room temperature for 45 min. After blocking, cells transfected with VLDLR WT or VLDLR p.(P565Q) and cotransfected with GFP‐HRas were incubated with mouse monoclonal anti‐HA‐tag primary antibody (1:200; Cell Signaling Technologies; Danvers, Massachusetts, United States) alone. Cells overexpressing VLDLR WT or p.(P565Q) only were incubated with both anti‐HA primary antibody and anticalnexin (an ER marker) rabbit monoclonal antibodies (1:200; Cell Signaling Technologies; Danvers, Massachusettes, United States) in 1% BSA for 45 min at room temperature.

After primary antibody incubation, cells were washed three times with 1X PBS and incubated with Alexa Fluor 555 antimouse secondary antibody (1:1000; Invitrogen) for HA and Alexa Fluor 488 antirabbit secondary antibody (1:1000; Invitrogen) for calnexin for 45 min at room temperature. Following secondary antibody incubation, cells were washed three times with 1X PBS, mounted on sterile slides using immunofluor mounting medium (ICN Biomedicals), and analyzed using a Nikon Eclipse confocal microscope (Nikon Instruments Inc.) with FITC and TRITC filters. Images were captured using a 100× oil immersion objective lens, modified and merged using ImageJ (Fiji) software. All images presented are single sections in the z‐plane.

Colocalization between HA‐tagged WT VLDLR or VLDLR missense variant p.(P565Q) and GFP‐HRas/calnexin was performed using the colocalization threshold plugin (Coloc2) on ImageJ (Fiji) software. Merged images were split, converted to 8‐bit format, and at least 35 cells were traced manually using the ROI manager for each corresponding channel. Pearson′s correlation coefficient was calculated between both channels. *R* values ranged from −1 to 1, where *R* = 1 indicates a positive correlation, *R* = 0 indicates no correlation, and *R* = −1 indicates a negative correlation between channels. *R* values are represented in box plots, and statistical analysis was performed using SigmaPlot 12.0 software, as represented in (Figure 3e, 3f) for GFP‐HRas and for calnexin, respectively.

Figure 3HeLa cells transiently transfected with HA‐tagged VLDLR wild‐type (panel a) or mutant p.(P565Q) expression construct and cotransfected with GFP‐HRas plasmid (localizes at the inner leaflet of the plasma membrane) were probed with anti‐HA primary antibody and fluorescently stained in red with Alexa Fluor 555 as illustrated in panels (a and b), and the merged images in the third column demonstrate colocalization with GFP‐tagged HRas. Moreover, HeLa cells exogenously expressing VLDLR wild type or the p.(P565Q) missense variant only were costained with the ER marker calnexin, as illustrated in panels (c and d). The merged images in the third column demonstrate colocalization with calnexin. All images were captured using the Nikon Eclipse system (Nikon Instruments Inc., Tokyo, Japan) equipped with FITC and TRITC filters. Images were captured using a 100× oil‐immersion objective lens. Images were modified, and a scale bar was added using ImageJ (Fiji) software. Scale bar = 30 *μ*m. Box plots representing Pearson′s correlation coefficients (n ≥ 35 cells) generated in SigmaPlot 12.0 for colocalization of VLDLR wild type and p.(P556Q) with (e) GFP‐HRas and (f) calnexin. *R* = 1 indicates a positive correlation, *R* = 0 indicates no correlation, and *R* = −1 indicates a negative correlation between channels.

**Figure:**
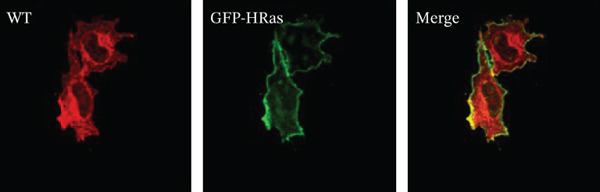
(a)

**Figure:**
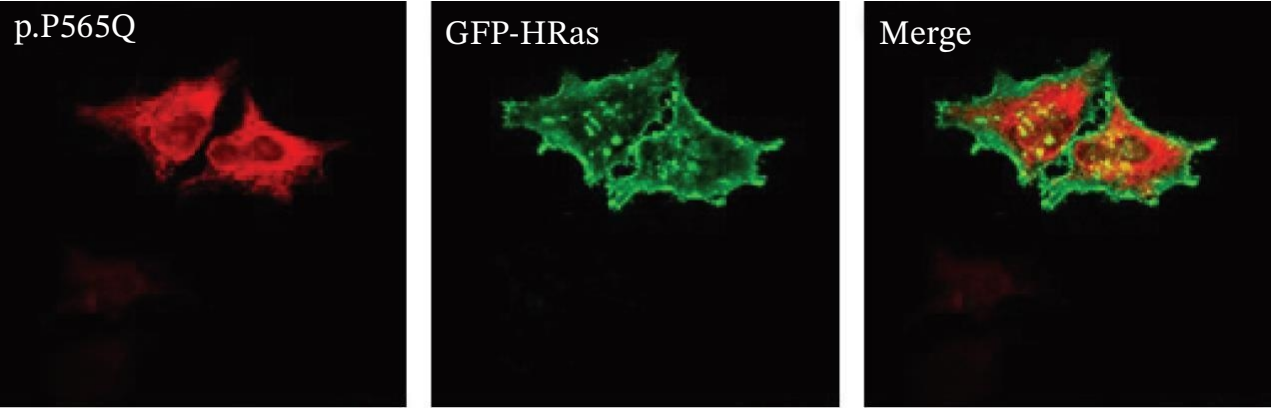
(b)

**Figure:**
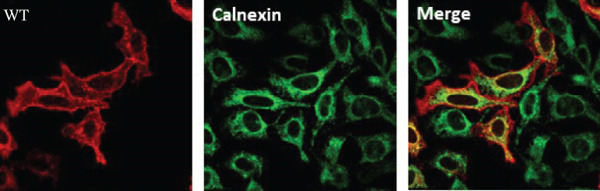
(c)

**Figure:**
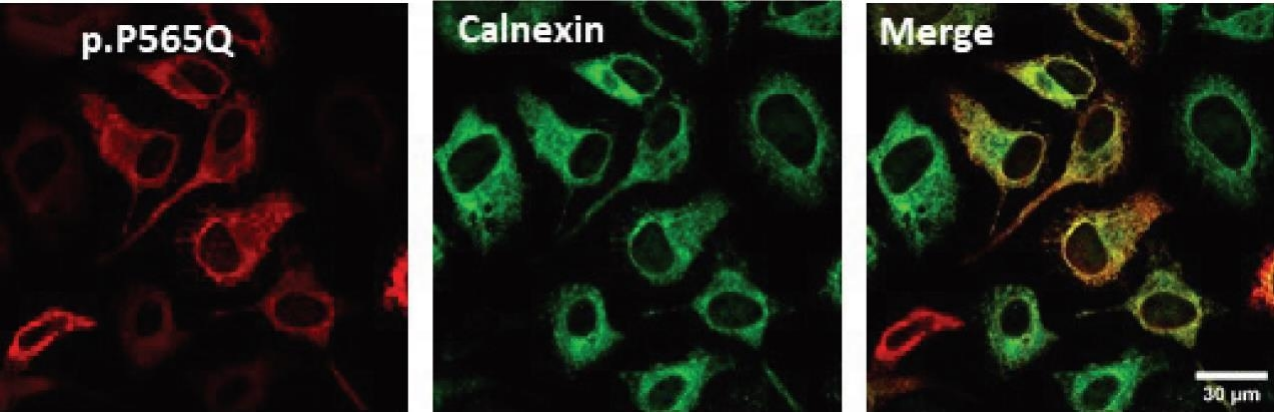
(d)

**Figure:**
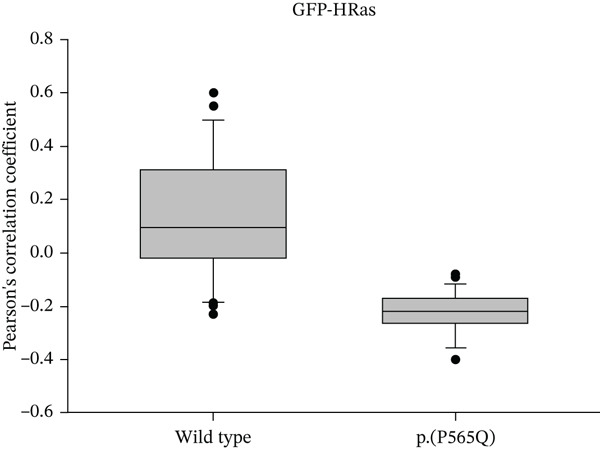
(e)

**Figure:**
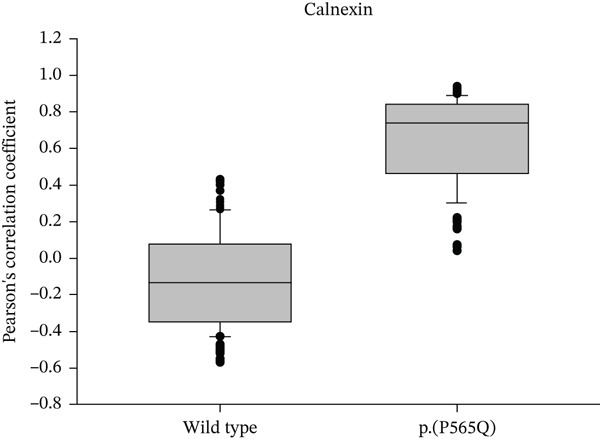
(f)

### 2.7. Endo H Sensitivity and Resistance Assay and Western Blot Analysis

Forty‐eight hours posttransfection, HEK293T cells overexpressing VLDLR WT or VLDLR p.(P565Q) mutant expression construct were harvested and centrifuged at 500 g for 5 min. Pellets were lysed with a 1× protease inhibitor (Halt protease inhibitor cocktail; Thermo Fisher Scientific, Waltham, Massachusetts, United States). Total protein concentration was calculated using a bicinchoninic acid protein assay (BCA kit; Thermo Fisher Scientific Pierce, Waltham, Massachusetts, United States). For the Endo H assay, following the manufacturer′s instructions (Sigma‐Aldrich, United States), 60 *μ*g of total protein was prepared with reaction and denaturation buffers. Boiled at 98°C for 5 min on the heat block and split into two equal aliquots of 30 *μ*g each, where one was treated with 100 U of Endo H enzyme, whereas the other served as an untreated control. Both aliquots were incubated at 37°C for 3.5 h. They were prepared in 4X Laemmli buffer (BioRad, Hercules, California, United States) and RIPA Lysis and Extraction Buffer (Thermo Fisher Scientific Pierce, Waltham, Massachusettes, United States) for western blot analysis.

The prepared samples were resolved on SurePAGE Precast Gels (GenScript, China) blotted onto poly (vinylidene difluoride) membrane (Thermo Fisher Scientific) and blocked with 5% BSA/1X TBST for 45 min at room temperature. After blocking, the membrane was incubated overnight at 4°C with monoclonal mouse anti‐HA primary antibody (1:4000; Cell Signaling Technologies) and mouse monoclonal anti–*β*‐actin primary antibody (1:1000; Santa Cruz Biotechnology, California, United States) diluted in 5% BSA/1X TBST. After primary antibody incubation, the PVDF membrane was washed three times with 1X TBST for 10 min each, probed with secondary antimouse antibody in 1X TBST for 45 min at room temperature, washed three times with 1X TBST for 10 min each, and developed using Enhanced Chemiluminescence Plus reagent (Thermo Fisher Scientific Pierce, Waltham, United States) and visualized on Typhoon FLA 9500 Imager (GE Healthcare Biosciences, Piscataway, New Jersey, USA). Similarly, western blot analysis was conducted following the same protocol above to evaluate the degradation route of VLDLR in HEK293 and HRD1‐KO cell lines. VLDLR was detected using an anti‐HA primary antibody, and anti–*β*‐actin was used as a loading control to detect *β*‐actin. Immunoblots were developed using ECL Plus reagent and visualized under Azure Sapphire Biomolecular Imager. Densitometric analysis for the immunoblots was performed using ImageJ (Fiji) software, and statistical analysis was done on SigmaPlot 12.0 software.

## 3. Results

### 3.1. Clinical Features

The proband (III:1) presented with motor delay early in life, crawling at age 1 but never achieving independent ambulation. She has a moderate to profound intellectual disability and has never developed language. There is no history of epilepsy, hearing, or vision problems. In her early twenties, she displayed marked truncal ataxia, characterized by a quadrupedal gait, and was able to stand and ambulate only with bilateral support, adopting a wide‐based gait (Figure 1c). Examination revealed mild nystagmus, poor limb coordination during alternate movements, and lower limb pyramidal signs, including bilateral Babinski sign, increased reflexes, mild spasticity, and clonus. Mild hypotonia was present, predominantly distally. She was able to follow simple commands but was unable to produce any language. The other affected siblings (Table 1 and Figure 1c) presented with a similar overall phenotype, with minor variations. Individual III:2 showed convergent strabismus and normal reflexes in the lower limbs, whereas Individuals III:4 and III:6 did not exhibit nystagmus.

### 3.2. Genetic Analysis

Whole exome sequencing of Individual III:1 identified a homozygous missense variant in *VLDLR* (NM_003383.5) (c.1694C > A; p.P565Q) (Figure 2a). The identified variant was absent from population databases (gnomAD 4.0) and classified as Variant of Uncertain Significance (VUS) per ACMG guidelines (PP3 strong, PM2 supporting, and BP1 supporting). Sanger sequencing confirmed the identified variant and its segregation with the disease phenotype within the family (Figure 2b, 2c).

### 3.3. In Silico Investigation of p.(P565Q)

Sequence conservation analysis reveals that Pro565 is fully conserved across mammals, as seen in Figure S2. This amino acid residue is located within the YWTD module III of the *β*‐propeller domain, specifically in a linker region connecting two beta strands (Figure 2d). Examination of the predicted 3D protein model (AF‐P98155‐F1‐v4) indicates that the p.(P565Q) substitution may disrupt protein structure by introducing a more flexible polar side chain that lacks the rigid cyclic structure of proline, thus compromising the stabilizing interactions Pro565 forms with neighboring residues. Most in silico tools predict p.(P565Q) as pathogenic, though some returned uncertain results (Table 2). The assessment of the stability and flexibility with DynaMut2 showed that the missense change is predicted to destabilize the protein structure (*ΔΔ*GStability −1.1 kcal/mol). To validate computational predictions, we conducted detailed cellular and biochemical studies of the p.(P565Q) variant to investigate its pathogenicity and underlying molecular mechanisms.

**Table 2:** In‐silico analysis done on variant effect predictor (VEP) by ensemble.

Missense variant	SIFT	PolyPhen	FATHMM	Meta LR	Meta RNN	Meta SVM	Mutation assessor	Mutation taster	PROVEAN	Primate AI
c.1694 > A										
p.Pro55GIn	Deleterious (0)	Probably_damaging (1)	D, D	D	D, D	D	H, H	D, D,D	D, D	T

Abbreviations: D, deleterious; H, high; T, tolerated

### 3.4. Immunofluorescence and Confocal Microscopy Reveal Perinuclear Localization of p.(P565Q), Suggesting ER Retention

We investigated the subcellular localization of p.(P565Q) using immunofluorescence and confocal microscopy. The VLDLR WT predominantly localized to the plasma membrane in transiently transfected HeLa cells, as confirmed by colocalization with plasma membrane marker GFP‐HRas (Figure 3a). In contrast, p.(P565Q) exhibited perinuclear intracellular localization and did not colocalize with GFP‐HRas (Figure 3b), suggesting retention in the endoplasmic reticulum (ER). Costaining of HeLa cells overexpressing VLDLR WT or mutant constructs with calnexin, a well‐known ER marker, further confirmed its ER retention, as the p.(P565Q) variant demonstrated stronger colocalization with calnexin (Figure 3c) compared with WT (Figure 3d).

### 3.5. p.(P565Q) Is Retained in the ER and Is Sensitive to Endo H Enzyme

To further confirm ER retention, Endo H susceptibility was conducted to assess the susceptibility of p.(P565Q) to Endo H enzyme. Endo H cleaves immature N‐glycans from misfolded proteins retained in the ER, preventing their trafficking to the Golgi apparatus for posttranslational modifications. Correctly folded proteins with complex mature glycans are resistant to Endo H treatment. The immunoblot showed two migrating bands for VLDLR WT: a faster migrating band at 130 kDa (precursor or immature VLDLR) and a slower band (mature VLDLR) at 150 kDa. In contrast, only the immature band was observed for p.(P565Q), confirming its retention in the ER (Figure 4a, 4b). In addition, we observed a molecular weight shift in the precursor form for both WT and p.(P565Q) Endo H‐treated samples, indicating susceptibility to Endo H. The mature form of VLDLR WT remained unchanged in both Endo H‐treated and untreated samples, indicating resistance to Endo H. Overall, our findings suggest that p.(P565Q) disrupts VLDLR trafficking to the plasma membrane, leading to its retention in the ER, and therefore, these data further confirm its pathogenicity.

Figure 4(a) HEK293T cells exogenously expressing either HA‐tagged VLDLR or the p.(P565Q) mutant expression construct were subjected to an endoglycosidase H (EndoH) assay to analyze their glycosylation profiles. The cells were harvested, and cell lysates were divided into equal aliquots of 30 *μ*g each. One aliquot was treated with EndoH, labeled as (+), whereas the other aliquot was left untreated to serve as a control, labeled as (−). (b) VLDLR maturation levels were calculated and plotted into a bar chart, and statistical analysis was run on SigmaPlot 12.0 software. Error bars represent ±SEM of six independent experiments; One‐way ANOVA was used to calculate the statistical significance of the VLDLR p.(P565Q) variant versus the VLDLR wild type. *p* value was calculated using the Holm–Sidak method, which is represented as ( ^∗^) *p* ≤ 0.05; ( ^∗∗^) *p* ≤ 0.01; ( ^∗∗∗^) *p* ≤ 0.001.

**Figure:**
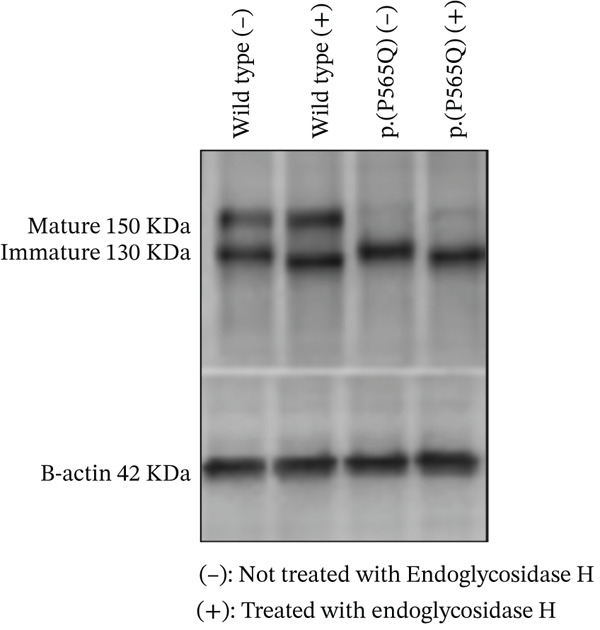
(a)

**Figure:**
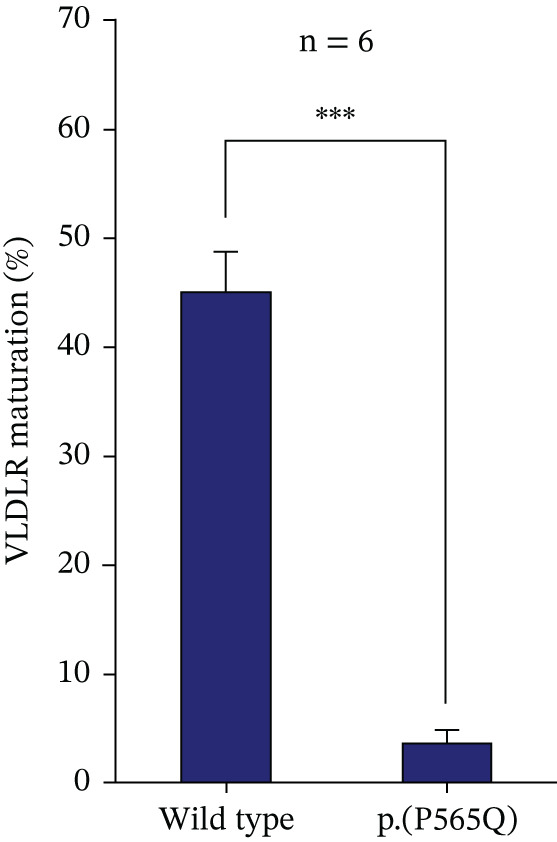
(b)

### 3.6. The p.(P565Q) Variant Is Likely Targeted for Degradation Through the Ubiquitin‐Mediated Proteasome Pathway

To better understand the ubiquitin‐mediated proteasome degradation of the VLDLR missense variant p.(P565Q). CRISPR‐Cas9‐generated HEK293 cells deficient in HRD1 E3 ubiquitin ligase HRD1 KO were transiently transfected with VLDLR WT or p.(P565Q) mutant expression constructs. Protein lysates were prepared, resolved into SDS‐PAGE gels, and analyzed by western blot analysis. Our results revealed a marked and significant accumulation of p.(P565Q) in HRD1 KO cells compared with its respective controls, VLDLR WT in HRD1‐KO, and p.(P565Q) in HEK293 cells, as shown in (Figure 5a, 5b). Our findings indicate that p.(P565Q) most likely undergoes degradation through the ubiquitin‐mediated proteasome pathway.

Figure 5(a) CRISPR‐Cas9‐generated HEK293 cells deficient in HRD1 E3 ubiquitin ligase and HEK293 (parental cell line) exogenously expressing HA‐tagged VLDLR wild type or the VLDLR missense variant p.(P565Q) were harvested, prepared, and analyzed by western blot analysis to detect accumulation of VLDLR in HRD1‐KO cells. (b) The relative protein to parental cells (%) was calculated for five independent experiments, normalized with *β*‐actin used as a loading control, and plotted into a bar graph using SigmaPlot 12.0 software. The statistical significance of the following comparisons was evaluated for: wild‐type HEK293 versus wild‐type HRD1‐KO, p.(P565Q) HEK293 versus (P565Q) HRD1‐KO, and wild‐type HRD1‐KO versus p.(P565Q) HRD1‐KO. Significance was represented as ( ^∗^) *p* ≤ 0.05; ( ^∗∗^) *p* ≤ 0.01; ( ^∗∗∗^) *p* ≤ 0.001.

**Figure:**
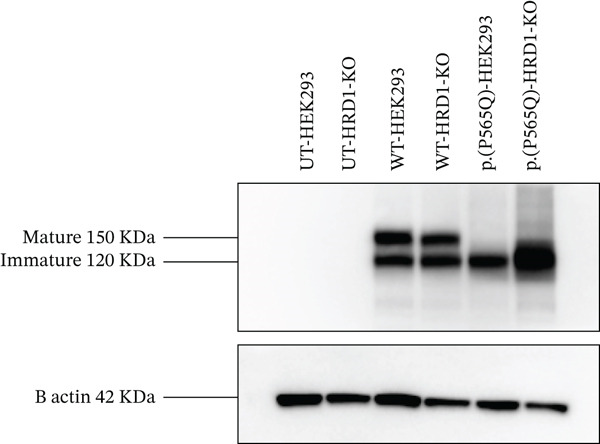
(a)

**Figure:**
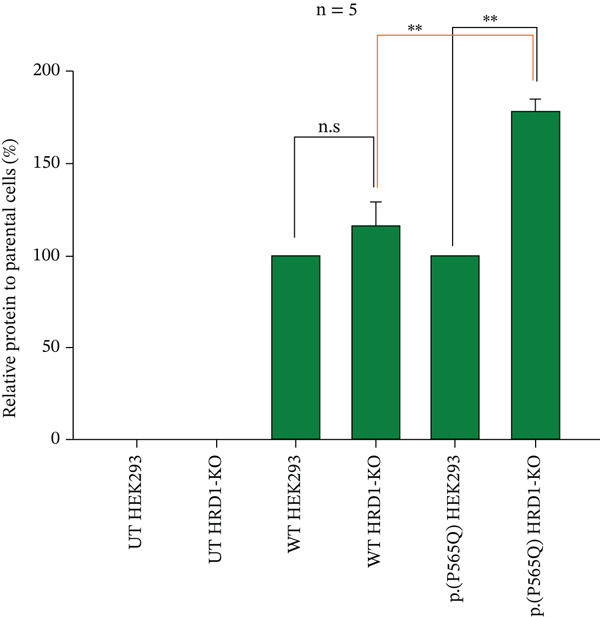
(b)

## 4. Discussion

This study broadens the genetic and geographical spectrum of CAMRQ1 by reporting the first family from the African continent associated with a novel missense loss‐of‐function variant p.(P565Q) in the *VLDLR* gene. Our biochemical validation studies confirmed that ER retention is likely the underlying pathogenic disease mechanism, reinforcing VLDLR′s pivotal role in reelin signaling pathways and brain development. CAMRQ1 is a rare autosomal recessive disorder characterized by ataxia associated with intellectual disability, delayed ambulation and speech, and cerebellar hypoplasia. Initial studies identified nonsense variants in the *VLDLR* gene associated with CAMRQ1 [2, 6, 35–37]. Later, several missense variants have been reported, largely in the *β*‐propeller domain and its neighboring EGF‐like repeats [2, 14, 33, 34, 38]. Given the clustering of mutations around the *β*‐propeller domain (Figure 2a), it is likely that the *β*‐propeller region in the EGF homology domain is less tolerant to variation. The phenotype of patients reported to date seems relatively uniform across reports, except for a recent case study of two siblings presenting with a neurodevelopmental disorder without cerebellar hypoplasia. Here, both individuals carried a homozygous nonsense variant (c.376C > T; p.(Gln126∗)) in the alternatively spliced Exon 4 in the VLDLR protein, which may have partially compensated for a milder phenotype of cerebellar hypoplasia [14]. Overall, no clear phenotype–genotype correlation has been reported for CAMRQ1, supporting the idea that brain abnormalities resulting from VLDLR deficiency are generally caused by a loss‐of‐function mechanism that impairs reelin signaling.

VLDLR is a transmembrane receptor critical for reelin‐mediated neuronal migration. Like other LDL receptor family members, VLDLR undergoes strict folding and quality control within the ER. HRD1 is an E3 ubiquitin ligase extensively studied for its role in eliminating misfolded proteins, and it is a core component of the retrotranslocon channel in the ERAD pathway. Aberrant proteins that fail to attain their native conformation are recognized by ERAD, retrotranslocated, ubiquitinated, and degraded by the proteasome [39, 40]. Our study showed that p.(P565Q) is retained in the ER and showed marked accumulation in HRD1‐KO cells, indicating that HRD1 plays a role in its degradation. As a result, this alters the intracellular trafficking of VLDLR and prevents its transport to the plasma membrane, which is necessary for receptor‐mediated endocytosis of extracellular ligands such as reelin [34]. In a previous study, the molecular characteristics of CAMRQ1‐causing VLDLR variants p.D487Y, p.D521H, and p.C706F, which were also found to be retained in the ER for extended durations, exhibited slower degradation compared with canonical ERAD substrates and abnormal cellular trafficking [34]. In another study, Kizhakkedath et al. [33] demonstrated the role of ubiquitin E3‐ligase SEL1L in mediating the degradation of these variants, which were susceptible to Endo H and Triton‐X solubility assays. These assays further confirmed the retention and aggregation of the VLDLR missense variants, which in turn activated the unfolded protein response (UPR), resulting in increased XBP‐1 mRNA levels. Further analysis with proteasomal (MG132, ALLN, and lactacystin) and lysosomal (leupeptin and NH4Cl) inhibitors showed that the accumulation of the VLDLR variants was more prominent with proteasomal inhibitors, suggesting that these variants are predominantly targeted for degradation by ubiquitin–proteasome‐mediated degradation. These findings align with our results for p.(P565Q), which accumulated in HRD1‐KO cells. Immunoprecipitation experiments confirmed interactions between the VLDLR variants and calnexin, as well as SEL1L, suggesting prolonged interactions in both of them, which contribute to ER stress [33, 34]. ERAD mechanisms have been reported for other members of the low‐density lipoprotein receptors, in particular LDLR, and its missense variants p.D445E, p.D482H, p.D622G, p.C667F, and p.R744Q associated with familial hypercholesterolemia (FH), were shown to accumulate upon treatment with MG132, indicating that these missense variants are possibly targeted for degradation via the ubiquitin‐proteasome degradation pathway [41, 42].

There is currently no targeted therapy for CAMRQ1; however, there is a growing interest in targeting the ERAD pathway as a therapeutic strategy for disorders caused by protein misfolding and retention. Emerging approaches aim to enhance protein folding, prevent excessive ER stress, and maintain ER homeostasis [39, 43]. Some of these therapies include chemical chaperones such as bortezomib, a proteasome inhibitor that blocks the delivery of misfolded proteins to the retrotranslocon for ubiquitination and subsequently prevents their degradation by the proteasome [44]. In addition, mTOR inhibitors like rapamycin and chemical chaperones such as tauroursodeoxycholic acid (TUDCA) and sodium 4‐phenylbutyrate (4‐PBA) have been shown to reduce ER stress and stimulate autophagy, with potential neuroprotective applications in neurodegenerative diseases [45, 46] and traumatic brain injury [47]. These approaches could potentially have applications for CAMRQ1, given the ER retention and high levels of VLDLR protein accumulated in HRD1‐KO cells observed for the p.(P565Q) VLDLR missense variant.

## 5. Conclusions

This study highlights the first description of disequilibrium syndrome in the African continent, thereby expanding the geographical and genetic spectrum of CAMRQ‐related disorders. We identified a novel missense variant p.(P565Q) in the *VLDLR* gene, which disrupts protein trafficking by causing retention in the ER. Our findings contribute to a deeper understanding of the pathogenesis of CAMRQ1 and underscore the importance of ERAD pathways in the disease. Further research into ER stress modulation could pave the way for future treatments for CAMRQ1 and related neurodevelopmental disorders, supporting the development of novel therapeutic interventions aimed at restoring VLDLR′s function by targeting its trafficking and degradation pathways. Additionally, this study highlights the importance of functional validation studies in confirming the pathogenicity of variants of uncertain significance, thereby ensuring more accurate diagnoses for affected individuals in underrepresented populations.

## Author Contributions

A.A.J.: methodology, writing—original draft, formal analysis, investigation, and visualization. A.S‐V.: methodology, analysis of interpretation of data, and revision of the manuscript for content. H.S.: revision of the manuscript for content. A.A‐M.: methodology and investigation. R.N.: revision of the manuscript for content. A.G.D.: study concept or design and revision of the manuscript for content. M.N.: study concept or design and revision of the manuscript for content. B.R.A.: formal analysis, supervision, funding, review and editing. P.M.R.C.: study concept or design, data collection, revising the article, and final approval.

## Funding

This study was supported by UAEU Zayed Center (31R232 12R073); “la Caixa” Foundation (10.13039/100010434ID 100010434); European Union′s Horizon 2020 research and innovation programme under the Marie Skłodowska‐Curie Grant Agreement (No. 847648, LCF/BQ/PI21/11830012); Research Seed Money Grant from the National Ataxia Foundation (1249136); UAEU PhD fellowship; and La Caixa Postgraduate Abroad fellowship. Open access publishing facilitated by United Arab Emirates University, as part of the Wiley ‐ United Arab Emirates University agreement.

## Ethics Statement

Ethics approval was granted from Comité National d′Éthique pour Ia Recherche en Santé (CNERS: SEN2021/26).

## Consent

Consent for publication was obtained from all participants.

## Conflicts of Interest

The authors declare no conflicts of interest.

## Supporting Information

Additional supporting information can be found online in the Supporting Information section.

## Supporting information


**Supporting Information 1** Figure S1: Sanger sequencing following site‐directed mutagenesis to confirm the p.(P565Q) VLDLR variant was successfully created. Clustal Omega alignment with the wild‐type VLDLR transcript (NM_003383.5) was used to validate the successful introduction of the amino acid substitution.

**Supporting Information 2** Figure S2: Sequence conservation analysis (genus_species|gene|UniProt entry ID) demonstrates conservation of the Pro565 residue in the VLDLR gene across the following 12 species: *Sus scrofa* (pig), *Canis lupus familiaris* (dog), *Jaculus jaculus* (Lesser Egyptian jerboa), *Panthera leo* (lion), *Mus musculus* (mouse), *Rattus norvegicus* (rat), *Pan troglodytes* (chimpanzee), *Bos taurus* (cow), *Oryctolagus cuniculus* (rabbit), *Sciurus vulgaris* (Eurasian red squirrel), *Homo sapiens* (human), and Rhinopithecus roxellana (Golden snub‐nosed monkey).

**Supporting Information 3** Table S1: Summary of reported cases of cerebellar ataxia, mental retardation, and disequilibrium syndrome (CAMRQ) in literature and their characteristics.

**Supporting Information 4** Table S2: Estimated prevalence of *CAMRQ*‐related disorders based on reported cases in the literature.

**Supporting Information 5** Table S3: p.(P565Q) mutagenic primers designed on PrimerX.

## Data Availability

Data available on request from the authors.
