# Supplementary material for: Identification of a Novel VLDLR Variant in the First Report of CAMRQ1 From Africa: Expanding the Spectrum of Cerebellar Ataxia Syndromes
Source: Hum Mutat. 2026 Apr 27;2026:4661238. doi: 10.1155/humu/4661238 (PMC13112595; doi:10.1155/humu/4661238)
Supplement: Supplementary file 1 — Supporting Information 1 Figure S1: Sanger sequencing following site‐directed mutagenesis to confirm the p.(P565Q) VLDLR variant was successfully created. Clustal Omega alignment with the wild‐type VLDLR transcript (NM_003383.5) was used to validate the successful introduction of the amino acid substitution. [file HUMU-2026-4661238-s004.pptx]

## Slide 1
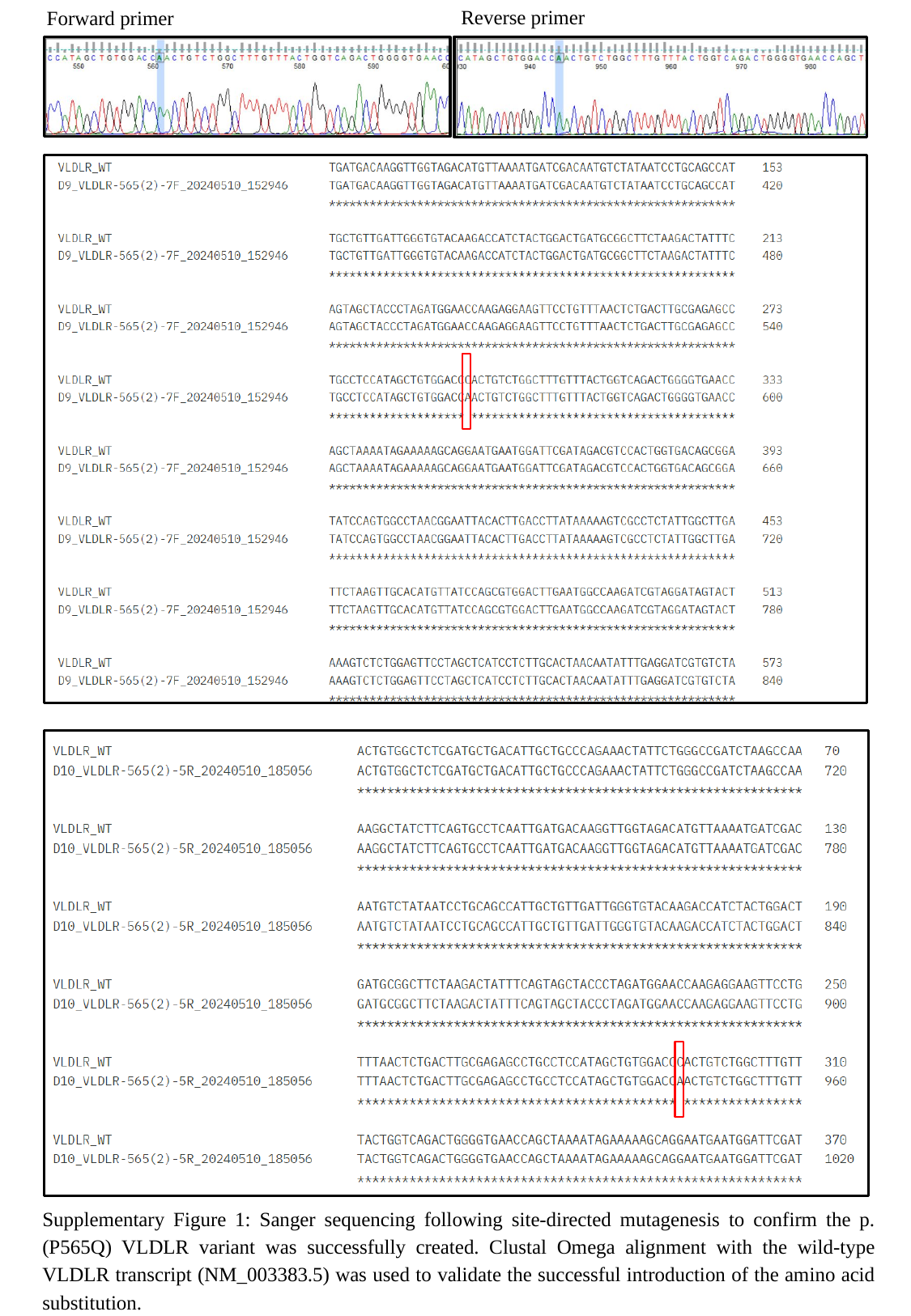

Reverse primer
Forward primer
Supplementary Figure 1: Sanger sequencing following site-directed mutagenesis to confirm the p.(P565Q) VLDLR variant was successfully created. Clustal Omega alignment with the wild-type VLDLR transcript (NM_003383.5) was used to validate the successful introduction of the amino acid substitution.
