# Supplementary material for: Identification of a Novel VLDLR Variant in the First Report of CAMRQ1 From Africa: Expanding the Spectrum of Cerebellar Ataxia Syndromes
Source: Hum Mutat. 2026 Apr 27;2026:4661238. doi: 10.1155/humu/4661238 (PMC13112595; doi:10.1155/humu/4661238)
Supplement: Supplementary file 2 — Supporting Information 2 Figure S2: Sequence conservation analysis (genus_species|gene|UniProt entry ID) demonstrates conservation of the Pro565 residue in the VLDLR gene across the following 12 species: Sus scrofa (pig), Canis lupus familiaris (dog), Jaculus jaculus (Lesser Egyptian jerboa), Panthera leo (lion), Mus musculus (mouse), Rattus norvegicus (rat), Pan troglodytes (chimpanzee), Bos taurus (cow), Oryctolagus cuniculus (rabbit), Sciurus vulgaris (Eurasian red squirrel), Homo sapiens (human), and Rhinopithecus roxellana (Golden snub‐nosed monkey). [file HUMU-2026-4661238-s003.pptx]

## Slide 1
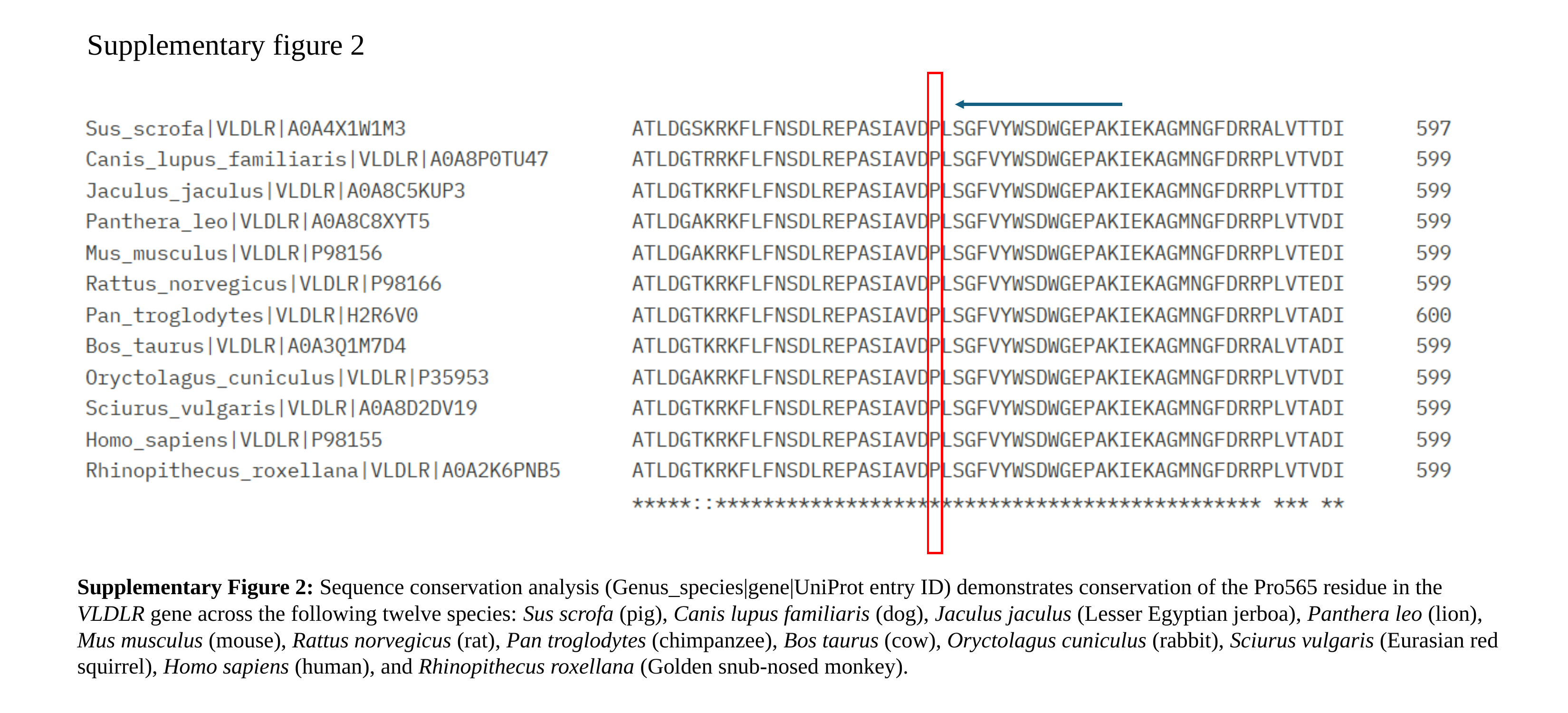

Supplementary figure 2
Supplementary Figure 2: Sequence conservation analysis (Genus_species|gene|UniProt entry ID) demonstrates conservation of the Pro565 residue in the VLDLR gene across the following twelve species: Sus scrofa (pig), Canis lupus familiaris (dog), Jaculus jaculus (Lesser Egyptian jerboa), Panthera leo (lion), Mus musculus (mouse), Rattus norvegicus (rat), Pan troglodytes (chimpanzee), Bos taurus (cow), Oryctolagus cuniculus (rabbit), Sciurus vulgaris (Eurasian red squirrel), Homo sapiens (human), and Rhinopithecus roxellana (Golden snub-nosed monkey).

## Slide 2
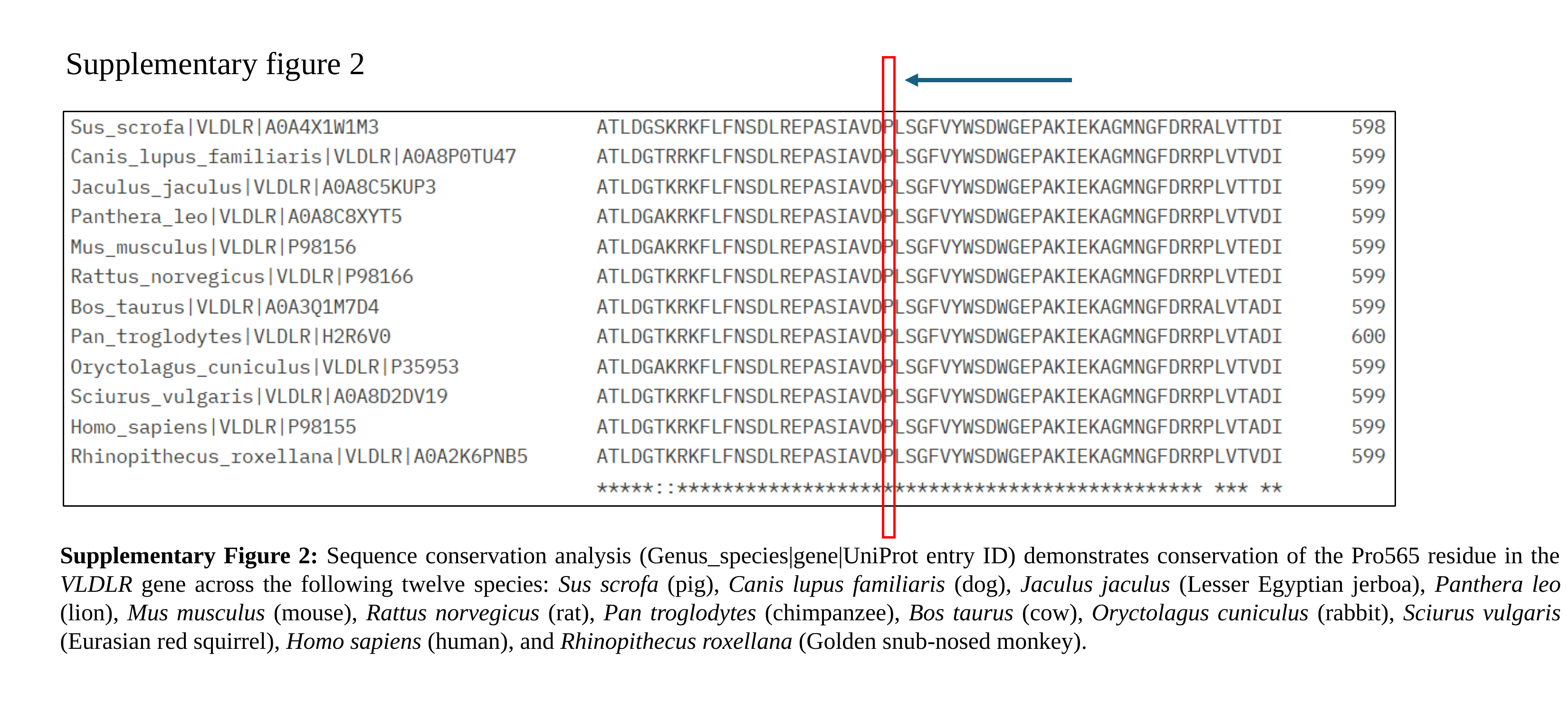

Supplementary figure 2
Supplementary Figure 2: Sequence conservation analysis (Genus_species|gene|UniProt entry ID) demonstrates conservation of the Pro565 residue in the VLDLR gene across the following twelve species: Sus scrofa (pig), Canis lupus familiaris (dog), Jaculus jaculus (Lesser Egyptian jerboa), Panthera leo (lion), Mus musculus (mouse), Rattus norvegicus (rat), Pan troglodytes (chimpanzee), Bos taurus (cow), Oryctolagus cuniculus (rabbit), Sciurus vulgaris (Eurasian red squirrel), Homo sapiens (human), and Rhinopithecus roxellana (Golden snub-nosed monkey).
