# Supplementary material for: Identification of a Novel VLDLR Variant in the First Report of CAMRQ1 From Africa: Expanding the Spectrum of Cerebellar Ataxia Syndromes
Source: Hum Mutat. 2026 Apr 27;2026:4661238. doi: 10.1155/humu/4661238 (PMC13112595; doi:10.1155/humu/4661238)
Supplement: Supplementary file 3 — Supporting Information 3 Table S1: Summary of reported cases of cerebellar ataxia, mental retardation, and disequilibrium syndrome (CAMRQ) in literature and their characteristics. [file HUMU-2026-4661238-s002.docx]

| **CAMRQ group** | **Gene** | **Main related pathways** | **Approximate number of reported cases in the literature** | **Common phenotypic features** | **Phenotypic hints of each subtype that can help differentiation** |
| --- | --- | --- | --- | --- | --- |
| CAMRQ-1 | *VLDLR* | - VLDL-triglyceride metabolism  - Reelin signaling pathway | 66 (≈70) | Delayed psychomotor development  Cognitive impairment  Cerebellar ataxia  Possible quadrupedal gait  Poor speech development  Cerebellar hypoplasia or atrophy on MRI | Cortical morphological abnormalities (cortical gyral simplification, pachygyria)  Cataracts, postnatal |
| CAMRQ-2 | *WDR81* | - [Signaling by Rho GTPases](https://pathcards.genecards.org/card/signaling_by_rho_gtpases)  - [RAC1 GTPase cycle](https://pathcards.genecards.org/card/rac1_gtpase_cycle) | 11 (≈10) |  | Dysmorphic facial features (coarse face, wide and short nape of the neck)  Skeletal deformities  Hirsutism  Degenerative |
| CAMRQ-3 | *CA8* | PKC-gamma calcium signaling pathway in Purkinje cells | 27 (≈ 30) |  | Pyramidal signs (uncommon)  Movement disorders (Parkinsonism, Dystonia, Dyskinesia; uncommon)  Febrile or focal seizures (rare) |
| CAMRQ-4 | *ATP8A2* | - [Ion channel transport](https://pathcards.genecards.org/card/ion_channel_transport)  - Ion transport by P-type ATPases  - Transport of small molecules | 11 (≈ 10) |  | Hearing loss (uncommon)  Optic atrophy (uncommon)  Ptosis  Dyskinetic quadriplegia, chorea  Degenerative |

**Supplementary Table S1:** Summary of Reported Cases of Cerebellar Ataxia, Mental Retardation, and Dysequilibrium Syndrome (CAMRQ) in Literature and Their Characteristics. * Potential new CAMRQ subgroups suggested by two case reports in the literature.
