# Supplementary material for: Identification of a Novel VLDLR Variant in the First Report of CAMRQ1 From Africa: Expanding the Spectrum of Cerebellar Ataxia Syndromes
Source: Hum Mutat. 2026 Apr 27;2026:4661238. doi: 10.1155/humu/4661238 (PMC13112595; doi:10.1155/humu/4661238)
Supplement: Supplementary file 4 — Supporting Information 4 Table S2: Estimated prevalence of CAMRQ‐related disorders based on reported cases in the literature. [file HUMU-2026-4661238-s001.docx]

**Supplementary Table S2: Data to generate Figure 1A (new potential candidate gene families not included):**

**Reported cases**

***VLDLR:***

11 American Hutterites

12 Canadian Hutterites

8 Iranian

16 Turkish (3+6+3+2+2)

5 Omani patients

10 Indian

4 Caucasian

***CA8:***

- 4 Iranian

- 1 Slovakia

- 1 Spain

- 2 Indian

- 1 Ukrainian

- 3 Turkish

- 3 Saudi

- 3 Pakistan

- 1 Iraqi

- 1 Caucasian

- 1 Syrian

- 6 Unknown

***WDR81:***

- 5 Turkish
- 4 Brazilian
- 2 Yemen

***ATP8A2:***

- 4 Turkish
- 3 Iranian
- 2 Unknown
- 2 Japanese

**If we divide these cases related to ancestry:**

1. **European (Caucasian): 31**

*VLDLR*: 11 American Hutterites,12 Canadian Hutterites, 4 Caucasian

*CA8*: 1 Slovakia, 1 Spain, 1 Ukrainian, 1 Caucasian

1. **Sub-Saharan African 0**
2. **Asian: 51**

*VLDLR*: 8 Iranian, 16 Turkish, 10 Indian

*CA8*: 4 Iranian, 2 Indian, 3 Turkish, 3 Pakistan

*WDR81*: 5 Turkish

*ATP8A2*: 4 Turkish, 3 Iranian, 2 Japanese

1. **Native American: 0**
2. **Oceanian (Melanesian, Polynesian, Micronesian): 0**
3. **Arab: 12**

*VLDLR*: 5 Omani patients

*CA8*: 3 Saudi, 1 Iraqi, 1 Syrian, 2 Yemen

1. **Indigenous Siberian and Central Asian**
2. **Admixed ancestry**

4 Brazilian


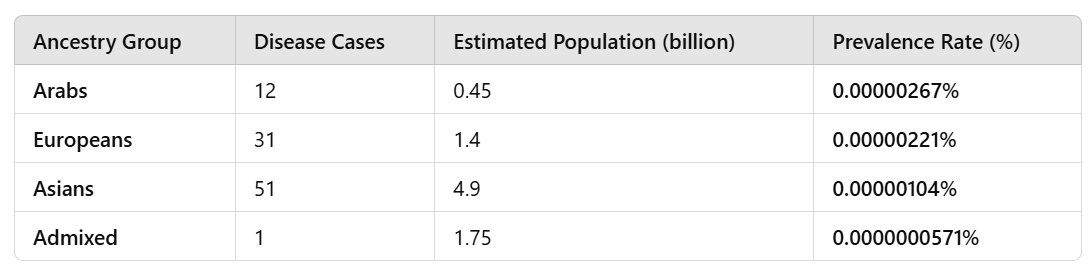


**Supplementary Table S2**: Estimated Prevalence of *CAMRQ*-Related Disorders based on reported cases in the literature.
