# Supplementary material for: Identification of a Novel VLDLR Variant in the First Report of CAMRQ1 From Africa: Expanding the Spectrum of Cerebellar Ataxia Syndromes
Source: Hum Mutat. 2026 Apr 27;2026:4661238. doi: 10.1155/humu/4661238 (PMC13112595; doi:10.1155/humu/4661238)
Supplement: Supplementary file 5 — Supporting Information 5 Table S3: p.(P565Q) mutagenic primers designed on PrimerX. [file HUMU-2026-4661238-s005.docx]

| **VLDLR missense variant** | **Primers** |
| --- | --- |
| **p.P565Q** | **Forward: 5' AGCTGTGGACCAACTGTCTGGCT 3'**  **Reverse: 5' AGCCAGACAGTTGGTCCACAGCT 3'** |

**Supplementary Table S3:** p.P565Q mutagenic primers designed on PrimerX
